# 

**Published:** 1892-01

**Authors:** 


### LIST OF CONTRIBUTORS.

Baker, S. S.
Bennett, E. E.
Billings, Frank S.
Breisacher, Leo.
Bryden, Williamson.
Buffington, G. L.
Cary, C. A.
Chase, J. M.
Clement, A. W.
Curtick, Cooper.
Dieckerhoff, Dr.
Dinwiddie, R. R.
Frame, D. P.
Francis, M.
Goodall, Theo. B.
Hamill, James.
Harger, S. J. J.
Hassel, Albert.
Hickman, R. W.
Hinebauch, T. D.
Johnson, Wyatt.
Kilbourne, F. L.
Knowles, M. E.
Lanigan, O. J.
Lothes, R.
Magill, C. H.
McCauley, H. R.
McCullaugh, Frank A.
McKinley, John S.
McNeil, J. C.
Miller, J. E.
McIntosh, D.
Meyers, J. C., Sr.
Moore, Veranus.
Norgaard, Victor A.
Nunn, J. A.
O'Leary, J.
Pearson, Leonard.
Pyle, Henry G.
Ridge, W. H.
Salmon, D. E.
Schwarzkopf, Olof.
Schweinitz, E. A. de
Scott, John.
Smith, Horace J.
Spencer, H. F.
Stanley, Edward.
Stiles, C. W.
Stinson, William.
Walley, Thomas.
Weber, S. E.
Webster, R. G.
Williams, W. L.
Williams, Charles.
Winchester, J. F.
Wilson, Matthew.
McFadyean, J.

### INDEX VOLUME XIII.

### A

PAGE
Abortion....... 670
About Cattle Ticks....... 1
Actinomycosis (Lumpy Jaw)....... 110
" ....... 161
" and U. S. Meat Inspection....... 177
" (a Consideration of)....... 269
" in Relation to Meat Inspection....... 351
" (a Consideration of)....... 358
Address (change of)....... 117
American Veterinary College Commencement....... 251
Animals (a Bacterial Disease of)....... 215
Aneurysms in Colic....... 355
Antiseptic Dressing of Wounds in the Lower Animals....... 193
Apology....... 251
Applications for Membership in the U. S. Veterinary Medical Association.. . 566
Are Aloes Admissible in Colics....... 67
Avian Tuberculosis (Review of)....... 429

### B

Bacterial (A Disease of Animals)....... 215
Baker S. S., D V.S., The Use of Lythium in Veterinary Practice....... 236
Bennett, E. E., F.R.C.V.S., A.V.D, Experiments with Mallein....... 53
Billings, Dr. F. S , A Consideration of Actinomycosis....... 269, 358
The Etiology of Southern Cattle Plague—Texas Fever, 397, 467, 526, 613, 676.
Bridges, George....... 120
Bryden, Williamson, V.S., Springhalt....... 12
Navicular Disease....... 210
Hoof Culture....... 344
Correction of a Clipping....... 509
Springhalt....... 569
Process of Refrigeration used for Dressed Beef in Transportation....... 666
Buffington, G., L., D.V.M., Sclerostoma Tetracanthum....... 734
Burke. R. W., F.R.C.V.S., Antiseptic Dressing of Wounds in the Lower Animals....... 193
Bureau of Animal Industry (The Scientific Investigation of the)....... 692

### C

Caicified Fibrous Nodes of the Liver and Lung of the Horse....... 149
California State Veterinary Medical Association....... 123, 261, 510

Carey, C. A —B.S., D.V.M., Aneurisms in Colic.......... .............. 355
A Peculiar Case of Ligation of the Small Intestines............. 427
Cattle Ticks (About)............................................. 1
Cattle Tuberculosis.............................................. 630
Change of Address........... ................................ 117
Chase, Dr. J. M., Paralysis .......... ...................... .. ..... 85
Chicago Veterinary College—Commencement............... ............. 258
Clement, A. W.—D.V.S., "So Called" Spinal Meningitis................ 486
Report of the Committee on Saniarty Science.... ..................... 742
Clinic—Traumatic Tetanus............................................. 37
Coal Oil Poisoning..................................................... 757
Colics (Are Aloes Admissible in)...................................... 67
College (Ontario Veterinary)........................................ 122
Colon (Malposition of the) ........................................ 499
Commencement—American Veterinary College........... ............... 253
" New York College of Veterinary Surgeons...................... 253
" Faculty of Comparative Medicine—McGill University... ... 255
" Ontario Veterinary College.... ........................ 258
Committee Report on Meat Inspection and Actinomycosis............ ..... 171
California State Veterinary Medical Association ........................ 776
Connecticut Veterinary Medical Association ............ .......... 125
Contribution to the Study of Mallein.......... . ..... .. ............ 160
Consideration of Actinomycosis (A)........................ ............269, 358
Contrasive Coloring in Horses.............. ........................ 507
Crib Biting...... .............. .......... ........................ 439
Cows (Parturient Apoplexy in).. .............................. ............ 16
Cow (Retention of a Dead Fœtus by a).................................. 142
Corn Stalk Disease.................. ... ........................... 83
Correction of a Clipping............................................... 509
Criticism (Extra Uerine Gestation)..................................... 429
Curtice, Cooper M.D., Vet., About Cattle Ticks................... .. ... 1
Parasites........... ........................ ............. 225

### D

Dieckerhoff, Dr., Contribution to the Study of Mallein.. ....................... 160
Diginity of a Professional Journal...................................... 116
Dinwidde, Dr. R. R., Some Parasitic Affections of Animals..,............ .. 342
Disease of Animals (A Bacterial).......... ........................... 215
Diseases of Rodents (Investigation upon the)............................. 374
Death of Henri F. Formad................ .. ............ ..... .... 447

### E

Etiology of Southern Cattle Plage—Texas Fever................. 397, 467, 676
Earth Worms of the Bacilli of Tuberculosis.................................. 629
Ear Vertigo in the Horse induced by Aspergillus Nigricans................ 247
Enlarged Mesenteric Gland and an Aneurism of the Anterior Mesenteric Artery. 425

### F

Faculty of Comparative Medicine McGill University—Commencement........ 255
Fibrous Ankylosis ... .. ................................................ 496

Foot and Mouth Disease... 185
Food Inspection... 658
Formad, Henri F., (Death of)... 447
Frame, D. P., Retention of a Dead Fœtus by a Cow ... 182
Francis, M., Notes on Parasites... 426

G

Giraffe (Tuberculosis in)... 181
Goodall, Thos. B.—F.R.C.V.S., Ear Vertigo in the Horse induced by Aspergillus Nigricans... 247

H

Harger, S. J. J.—V.M.D., Calcified Fibrous Nodes of the Liver and Lungs of the Horse... 149
Successful Aponeurotomy in Springhalt... 180
Recent Investigation on Tetanus... 453
Hamill, James, V S., Horse-shoeing—A Criticism... 315
Hæmorrhagie Anæmia in Cows... 326
Hickman, R. W. -V.M.D., Actinomycosis, (Lumpy Jaw)... 110
" " ... 161
Hinebauch, T. V.—M.S., V.S., Parturient Apoplexy in Cows... 18
Report of Cases of Cerebro-Spinal Meningitis... 327
Rheumatism in Horses... 725
Hippocrates (The Oath of)... 387
Hoof Culture... 344
Horses (Locoed)... 435
" (Importation of)... 491
" (Contractive Coloring in)... 507
" (Texas Mange) Porrigo... 426
Horse-shoeing a Criticism... 315
Horse Shows (Veterinary Inspection at)... 385

I

Illinois State Veterinary Medical Association... 58, 263, 724
Importation of Horses... 491
Indiana Veterinary Association... 515
Industry (The Bureau of Animal Scientific Investigation)... 692
Influenza... 21
Inspection of Veal (The)... 88
" National and International of Meat... 309
" Food... 658
Intestines (A Peculiar Case of Ligation of the Small)... 427
Is Osteoporosis Infectious... 244
Investigation upon the Diseases of Rodents... 374
Investigation on Tetanus (Recent)... 453
Iowa State Veterinary Medical Association... 724
" " " " " ... 780

J

Jaw Lumpy (Actinomycosis)... 110
" " ... 239
Johnson Wyatt, M.D., Local Tuberculous Abscess in in a Bull... 673
Journal (Dignity of a)... 116

### K

Kansas Veterinary Medical Association........................................ 267
Keystone Veterinary Medical Association ...................................... 138
" " " " .......................................... 449
" " " " .......................................... 723
" " " " .......................................... 723
Kilborne, F. L., B.V.S., The use of Mallein for the Diagnosis of Glanders in Horses, and Experiments with an Albumose extracted from Cultures of the Bacillus Malleus.......................... 643
Killock, Dr. J. ............................................................ 121
Knowles, M. E., D.V.S., Sterility of Mares.................................. 7

### L

Lanigan, Dr. O. J., Purulent Metritis........................................ 26
Ligation of the Small Intestines (A Peculiar Case of) ........................ 427
Locoed Horses................................................................ 435
Local Tuberculous Abscess in a Bull......................................... 673
Lump Shoulder................................................................ 90
Lumpy Jaw (Actinomycosis) ................................................ 110, 239
Lithium in Veterinary Practice (The use of)................................. 236

### M

Mallein, The use of, for the Diagnosis of Glanders in Horses, and Experiments with an Albumose extracted from Culture of the Bacillus Malleus ..........................................
" (Contribution to the Study of)........................................ 160
" (Experiments with).................................................... 643
" (Contribution to the Study of......................................... 91
Malpositions of the Colon.................................................... 499
Mares (Sterility of)......................................................... 7
Maryland State Veterinary Medical Association—Special Meeting................ 205
" " " " Sixth Annual Meeting....... 206
Massachusetts Veterinary Association, 62, 139, 202, 261, 393, 394, 395, 513, 776, 777
MaGill, C. H., V.M.D., The Inspection of Veal................................ 88
McCauley, H. R., V. S., Are Aloes Admissible in Colics....................... 67
McCleay, Sir Wm., Kt.F.L.S., M.L C........................................... 192
McCullaugh, Frank A., Dr., Locoed Horses..................................... 435
McFadyean, J., M.B., B.Sc., The Staining of Tubercle Bacilli in Milk......... 503
McGill University, Faculty of Comparative Medicine—Commencement.............. 255
McNeill, J. C.—V.M D., Is Osteoporosis Inflctious............................ 244
McIntosh, Dr. D., Parturient Apoplexy in Cows................................ 16
Medical Zoology (Review of Recent Publications in)........................... 101
Meat Inspection (The Committee Report on).................................... 107
" " " " " " .............................................. 171
Meeting of the United States Veterinary Medical Association.................. 489
Metritis (Purulent).......................................................... 26
Meyers, J. C., Sr., V.S., Tuberculosis in Giraffe............................ 181
Miller, J. E., M.R.C.V.S., Enlarged Mesenteric Gland and an Aneurism of the Anterior Mesenteric Artery........................................ 425

Moellers Surgery.................................................... 267
Montreal Veterinary Medical Association.............................. 63
Moore, Veranus A.—B.S., M.D., Mouse Scepticæmia Bacilli in a Pig's Spleen. 333
Mouse Scepticæmia Bacilli in a Pig's Spleen.......................... 333
Morris, Dr. Claude D., Tetanus....................................... 439
Mouth and Foot Diseases.............................................. 185

N

National and International Meat Inspection........................... 309
Navicular Diseases................................................... 210
Necrology............................................................ 192
New Jersey Veterinary and Medical Association........................ 330
New York State Veterinary and Medical Association.................... 127
" " " " " " "....................................................... 438
" " " " " " "....................................................... 761
Norgaard, Dr. Victor A., Actinomycosis............................... 161
North Eastern Iowa Veterinary Medical Association.................... 125
North Dakota State Veterinary Medical Association.................... 331
Notes on Parasites................................................... 65
" " "................................................................ 143
" " "................................................................ 319
" " "................................................................ 346
" " "................................................................ 426
" " "................................................................ 464
" " "................................................................ 517
New Jersey State Committee of Arrangements........................... 564
New Jersey Veterinary Medical Association............................ 639
New York State Veterinary Medical Society............................ 571
New York State and Tuberculosis in Cattle of the United States....... 721

O

Oath of Hippocrates (The)............................................ 387
Ohio State Veterinary Medical Association............................ 139
Ohio and Michigan State Veterinary Medical Association (Joint Session of).... 468
Ontario Veterinary College........................................... 122
" " "................................................................ 204
" " "................................................................ 250
" " Medical Association.............................................. 139
Opportunities (Neglected)—Agricultural Shows......................... 461
Osteoporis (Is it Infectious)........................................ 244

P

Paralysis............................................................ 85
Parasites, Notes on.................................................. 65
" ".................................................................. 143
" ".................................................................. 223
" ".................................................................. 465
" ".................................................................. 517
Patents, Recent...................................................... 38
" "................................................................... 118

Patents, Recent.......... 186
Parasitic in Pigs, Strongylus Rubidus, a New Species of Nematode.......... 207
Parturient Apoplexy in Cows..........16, 18
Peters, Dr. (Reply to).......... 28
Purulent Metritis.......... 26
Pearson, Leonard P. S., V.M.D., Tuberculosis of Cattle.......... 630
Pennsylvania State Medical Association.......... 641
" " " ".......... 264
Philadelphia Society of Veterinary Medicine.......... 140
" " " ".......... 450
Plague (The Etiology of Southern Cattle—Texas Fever)..........467, 526, 613
Professional Journal (Dignity of a).......... 116
Proceedings of the Association for the Study of Comparative Psychology, MaGill University, Montreal.......... 765
Publications (Review of Recent in Medical Zoology).......... 101
" " " " ".......... 557
Pyle, Dr. Henry G., D.V.S., A Bacterial Disease of Animals.......... 215

Q

Quittor or Quitter.......... 71

R

Recent Patents..........38, 118, 186
" Investigations in Tetanus.......... 453
Reply to Dr. Peters.......... 28
Report of Cases of Cerebro-Spinal Meningitis.......... 327
Report of the Committee on Sanitary Science, and Notice to the Pennsylvania State Veterinary Medical Association.......... 742
Report Committee on Diseases.......... 749
Resolutions of Respect.......... 761
Review of Avian Tuberculosis.......... 429
Review of Recent Publications in Medical Zoology.......... 557
Rheumatism in Horses.......... 725
Ridge, W. H., V.M.D., Hæmorrhagie Anæmia in Cow.......... 326
Rodents, (Investigation upon the Disease of).......... 374
Royal Veterinary College.......... 41
Rubidus Strongylus, A new Species of Nematode, Parasitic in Pigs.......... 207

S

Salmon, Dr. D. E., Reply to Dr. Peters.......... 28
The Scientific Investigation of the Bureau of Animal Industry.......... 692
Schwarzkopf, Olaf, Committee Reports on Meat Inspection and Actinomycosis. 171
Food Inspection.......... 658
Schweinitz, E. A. de., Ph.D., The use of Mallein for the Diagnosis of Glanders in Horse and Experiments with an Albumose Extracted from Cultures of the Baccillus Malleus.......... 643
Scientific Investigation of the Bureau of Animal Industry.......... 692
Sciatica.......... 242
Scott, John, Sciatica.......... 242
Scientific Honors.......... 191

Sclerostoma Tetracanthum........ 734
Shoulder Lump........ 90
Sheep (Worms in)........ 46
Shows (Veterinary Inspection at Horse)........ 385
Society for the Study of Comparative Psycology of McGill University, Montreal........ 135
"So Called" Spinal Meningitis........ 486
Some Parasitic Affections of Animals........ 342
Spencer, H. F., D.V.S., Corn Stalk Disease........ 83
Stanley, Edward, F.R.C.V.S., Worms in Sheep........ 46
Staining of Tubercle Bacilli in Milk (The)........ 503
Stiles, Chas. W., Ph.D., Notes on Parasites........ 65
" " " " " " "........ 143
" " " " " " "........ 319
" " " " " " "........ 346
" " " " " " "........ 517
Stiles, Review of Recent Publications in Medical Zoology........ 101
" " " " " " " "........ 557
Strongylus Rubidus, a new Species of Nematode Parasitic in Pigs........ 207
Springhalt........ 12
"........ 569
Sterility of Mares........ 7
Stinson, Wm., V.S., Shoulder Lump........ 90
Strongylus Armatus........ 579
Surgery, Moellers........ 267
Successful Aponeurotomy in Springhalt........ 180

### T

Tetanus (Recent Investigation on)........ 453
The Indiana Veterinary Association........ 515
The use of Mallein for the Diagnosis of Glanders in Horses and Experiments with an Albumose Extracted from Cultures of the Bacillus Malleus........ 643
Ticks, (About Cattle)........ 1
Traumatic Tetanus—Clinic........ 37
Tuberculosis........ 759
"........ 762
Tuberculosis in a Giraffe........ 181
" of Cattle........ 630
Two Cases of Albumen-Urea in Horses........ 562

### U

United States Veterinary Medical Association, Comitia Minora........ 45
" " " " " " "........ 117
United States Veterinary Medical Association........ 192
" " " " "........ 201
" " " " "........ 437
" " " " "........ 564
" " " " "........ 609
United States Meat Inspection and Actinomycosis........ 177

United States Army Veterinary Service ........................ 189
Use of Lythima in Veterinary Practice........................ 236
Use of Mallein for the Diagnosis of Glanders in Horses, and Experiments with an Albumose extracted from Cultures of the Bacillus Malleus (The)... 643

V

Variola and Vaccine........................ 184
Veal, (The Inspection of)........................ 88
Veterinarians as Educators........................ 490
Veterinary Inspection at Horse Shows........................ 385
Veterinary Medical Association of New Jersey........................ 639
Veterinary Colleges—They Come........................ 719

W

Warranty........................ 295
Walley, Thomas, M.R.C.V.S., Malpositions of the Colon........................ 499
Weber, S. E., V.S., Actinomycosis in relation to Meat Inspection........................ 374
Review of the Avian Tubercolosis........................ 429
Webster, R. G., V.M.D., Abortion........................ 670
Western Iowa Veterinary Medical Association........................ 203
" " " " "........................ 332
Williams, Charles, V.M.D., Quittor or Quitter........................ 71
Williams, W. L., V.S., Actinomycosis in relation to Meat Inspection........................ 171
" " " " " "........................ 351
Wilson, Matthew, M.R.C.V.S., Influenza........................ 21
Winchester, J. F., D.V.S., Strongylus Armatus........................ 579
Worms in Sheep........................ 46

Z

Zoology (Review of Recent Publications in Medical)........................ 101
" " " " "........................ 755

### ILLUSTRATIONS, VOL. XIII.

| Fig. | | Page. |
|---|---|---|
| 1 | Cervical Speculum | 11 |
| 2 | Vaginal Speculum | 11 |
| 3 | Applicator | 11 |
| 4 | Curette | 11 |
| 5 | Applicator Forceps | 11 |
| 6 | Vulcellum Forceps | 11 |
| 7 | Horseshoe | 38 |
| 8 | Udder-Protector | 38 |
| 9 | Fastening for Horse Blankets | 38 |
| 10 | Horse-Blinder | 38 |
| 11 | Horse-Collar | 39 |
| 12 | Horse-Tail Holder | 39 |
| 13 | Veterinary Instrument | 39 |
| 14 | Cattle-Guard for Railways | 39 |
| 15 | Ox-Collar | 39 |
| 16 | Feed-Trough | 39 |
| 17 | Time Stock-Feeder | 40 |
| 18 | Suture-Instrument | 41 |
| 19 | Feed-Trough | 40 |
| 20 | Myzomimus Scutatus | 67 |
| 21 | Quittor Shoe, La Fosse, Sr. | 79 |
| 22 | " " La Fosse, Jr. | 79 |
| 23 | " " Rey | 79 |
| 24 | " " Renault | 79 |
| 25 | Horseshoe | 118 |
| 26 | Portable Crib for Feeding Cattle and Hogs | 118 |
| 27 | Horseshoe-Nail Clincher | 118 |
| 28 | Horseshoe | 118 |
| 29 | Loop for Cow-Bells | 118 |
| 30 | Horse-Cleaner | 119 |
| 31 | Feed-Trough | 119 |
| 32 | Harness-Saddle | 119 |
| 33 | Expansible Horseshoe-Calk | 119 |
| 34 | Horse Blanket | 119 |
| 35 | Filaria Cervina | 144 |
| 36 | Calcified Fibrous Tubercle of the Lung | 158 |
| 37 | Calcified Fibrous Node of the Lung | 159 |
| 38 | Embolic Tubercles in a Glandered Lung | 159 |
| 39 | Actinomycosis | 161 |
| 40 | Actinomycosis in the Lower Lip of a Cow | 163 |
| 41 | Actinomycosis Developing | 164 |

42 Actinomycosis Centre........ 164

43 Actinomycosis in Postpharyngeal Gland........ 165

44 Tuberculosis in Postpharyngeal Gland........ 166

45 Marsh-Horseshoe........ 186

46 Collar-Pad........ 186

47 Horseshoe-Calk........ 186

48 Removable Horseshoe-Calk........ 186

49 Curry-Comb........ 187

50 Horseshoe........ 187

51 Bridle-Blinder........ 187

52 Horseshoe........ 187

53 Safety-Bridle and Relievable Bit........ 187

54 Nose-Ringing Tool........ 188

55 Crib for Feeding Cattle........ 188

56 Horseshoe........ 188

57 Sling-Cinch........ 188

58 Horseshoers' Tool........ 188

59 Caudal Portion of a Male........ 208

60 Chitinous Support........ 208

61 Spicules Isolated........ 208

62 Sporozoa, (Coccidium Perforans)........ 320

63 Coccidium Oviforme of Rabbits........ 322

64 Esmarch Tube........ 338

65 The Lungs........ 375

66 The Lungs........ 377

67 Portion of Jejunum........ 379

68 The Cæcum and Portion of Colon........ 381

69 Annular Constriction of Cæcum........ 382

70 Tubercular Nodules........ 383

71 Left Lobe of Liver........ 385

72 Tubercular Enlargement........ 431

73 Right and Hind Quarter and Abdominal Wall........ 483

74 A Portion of a Villus of a Dog's Intestines........ 518

75 Various Stages in the Development of Coccidium Bigeminum........ 518

76 Type Figure........ 521

77 Anterior Portion of Mermis Crassa........ 523

78 Mouth and Papillæ Highly Magnified........ 523

79 Tail of Male Mermis Crassa Magnified........ 524

80 End of Spicule, Showing Evaginated Canal........ 524

81 Diagramatic Representation of the Phases of the Development of the Texas Fever Germ........ 537

82 The Southern Cattle Plague Germ........ 538

83 The Swine Plague Germ........ 538

84 Bacilli........ 538

85 Strongylus Armatus........ 608b

Fig. I. Male; *a*, caudal pouch; *b*, spicula.

Fig. II. Female; *a*, vulva; *b*, anus.

Fig. III. *a*, capsule; *b*, longitudinal line or rib; *c*, anterior teeth; *d*, anterior cilia; *e*, papillæ; *f*, posterior papillary bodies or plates;

*g*, anterior armed pharyngeal ring; *h*, pharyngeal capsule; *k*, anterior constricted portion of œsophagus; *l*, posterior ventricle or bulb.
Fig. IV. *a*, anterior teeth; *d*, anterior cilia.
Fig. V. *b*, longitudinal line or rib; *f*, posterior plates.
Fig. VI. Anterior cilia.
Fig. VII. Anterior papillæ.
Fig. VIII. Fine anterior teeth.
Fig. IX. *a*, caudal pouch of male; *b*, spicula.
Fig. X. Posterior moiety of female; *b*, anus.
Fig. XI. Undeveloped or agamous worm, in thin transparent membrane, which is shed on reaching maturity.
Fig. XII. Ovum.
Fig. XIII. Male and female worms natural size, as attached during copulation.
86 Sclerostoma Equinum........608c
Fig. I. Caudal extremity of the male Sclerostoma equinum. (Neumann.)
Fig. II. Fragments of the cæcum of a horse, showing the tumors of different sizes due to the sclerostomes, as well as parasites fixed on the mucous membranes. (Neumann.)
87 Fig. I. Verminous aneurism of the great mesentric artery; one-half natural size. (Railliet.)........608d
Fig. II. Abdominal aorta of a horse with its ramifications. (Neumann.)
88 Rheumatism in Horses.
*a*, Protrusion of Os Calcis........727
*b*, Fiber of Gastrocnemius Externus........727
89 Depression on Articular Surface of Patella........729
90 *a*, Depression caused by Absorption of Articular Cartilage........731
*b*, Rupture of Popliteus from its Origin........731
*c*, Elevation and Depression the Result of Deposition........731
91 *b*, Absorption of Cartilage........732
*b*, Rupture of Origin of Popliteus Containing Particles of Bone........732
92 *a*, Denuded Bone Showing Result of Absorption of Cartilage........733

### UNITED STATES VETERINARY MEDICAL ASSOCIATION COMITIA MINORA.

A meeting of the Comitia Minora of the United States Veterinary Medical Association will be held at Hotel Royal, Sixth avenue and 40th street, New York, on Saturday, February 20th, 1892, 8 P. M.

Arrangements for the International Veterinary Congress at Chicago, 1893; and the place of meeting of the Association for 1892, will be considered, in addition to matters of routine business.

Suggestions and communications, to be for the Comitia Minora, should be submitted to the Secretary.

By Order of the President,

W. H. Hoskins, *Secretary*, R. S. Huidekoper.

12 South 37th street, Philadelphia.

